# Expansion of the Human Phenotype Ontology (HPO) knowledge base and resources

**DOI:** 10.1093/nar/gky1105

**Published:** 2018-11-22

**Authors:** Sebastian Köhler, Leigh Carmody, Nicole Vasilevsky, Julius O B Jacobsen, Daniel Danis, Jean-Philippe Gourdine, Michael Gargano, Nomi L Harris, Nicolas Matentzoglu, Julie A McMurry, David Osumi-Sutherland, Valentina Cipriani, James P Balhoff, Tom Conlin, Hannah Blau, Gareth Baynam, Richard Palmer, Dylan Gratian, Hugh Dawkins, Michael Segal, Anna C Jansen, Ahmed Muaz, Willie H Chang, Jenna Bergerson, Stanley J F Laulederkind, Zafer Yüksel, Sergi Beltran, Alexandra F Freeman, Panagiotis I Sergouniotis, Daniel Durkin, Andrea L Storm, Marc Hanauer, Michael Brudno, Susan M Bello, Murat Sincan, Kayli Rageth, Matthew T Wheeler, Renske Oegema, Halima Lourghi, Maria G Della Rocca, Rachel Thompson, Francisco Castellanos, James Priest, Charlotte Cunningham-Rundles, Ayushi Hegde, Ruth C Lovering, Catherine Hajek, Annie Olry, Luigi Notarangelo, Morgan Similuk, Xingmin A Zhang, David Gómez-Andrés, Hanns Lochmüller, Hélène Dollfus, Sergio Rosenzweig, Shruti Marwaha, Ana Rath, Kathleen Sullivan, Cynthia Smith, Joshua D Milner, Dorothée Leroux, Cornelius F Boerkoel, Amy Klion, Melody C Carter, Tudor Groza, Damian Smedley, Melissa A Haendel, Chris Mungall, Peter N Robinson

**Affiliations:** 1 Charité Centrum für Therapieforschung, Charité—Universitätsmedizin Berlin Corporate Member of Freie Universität Berlin, Humboldt-Universität zu Berlin, and Berlin Institute of Health, Berlin 10117, Germany; 2 Einstein Center Digital Future, Berlin 10117, Germany; 3 Monarch Initiative, monarchinitiative.org; 4 The Jackson Laboratory for Genomic Medicine, Farmington, CT 06032, USA; 5 Oregon Health & Science University, Portland, OR 97217, USA; 6 Genomics England, Queen Mary University of London, Dawson Hall, Charterhouse Square, London EC1M 6BQ, UK; 7 Environmental Genomics and Systems Biology, Lawrence Berkeley National Laboratory, Berkeley, CA 94720, USA; 8 European Bioinformatics Institute (EMBL-EBI), Wellcome Trust Genome Campus, Cambridge, UK; 9 Linus Pauling institute, Oregon State University, Corvallis, OR, USA; 10 William Harvey Research Institute, Queen Mary University College of London; 11 UCL Genetics Institute, University College of London; 12 UCL Institute of Ophthalmology, University College of London; 13 Renaissance Computing Institute, University of North Carolina at Chapel Hill; 14 Western Australian Register of Developmental Anomalies and Genetic Services of Western Australia, Department of Health, Government of Western Australia, WA, Australia; 15 School of Paediatrics and Telethon Kids Institute, University of Western Australia, Perth, WA, Australia; 16 Institute for Immunology and Infectious Diseases, Murdoch University, Perth, WA, Australia; 17 Spatial Sciences, Department of Science and Engineering, Curtin University, Perth, WA, Australia; 18 The Office of Population Health Genomics, Department of Health, Government of Western Australia, Perth, WA, Australia; 19 SimulConsult, Chestnut Hill, MA, USA; 20 Neurogenetics Research Group, Vrije Universiteit Brussel, Brussels, Belgium; 21 Pediatric Neurology Unit, Department of Pediatrics, UZ Brussel, Brussels, Belgium; 22 Garvan Institute of Medical Research, Darlinghurst, Sydney, NSW 2010, Australia; 23 Centre for Computational Medicine, Hospital for Sick Children and Department of Computer Science, University of Toronto, Toronto, Canada; 24 National Institute of Allergy and Infectious Diseases, National Institutes of Health, Bethesda, MD, USA; 25 Rat Genome Database, Department of Biomedical Engineering, Medical College of Wisconsin & Marquette University, 8701 Watertown Plank Road Milwaukee, WI 53226, USA; 26 Bioscientia GmbH, Ingelheim, Germany; 27 CNAG-CRG, Centre for Genomic Regulation (CRG), The Barcelona Institute of Science and Technology, Baldiri Reixac 4, Barcelona 08028, Spain; 28 Universitat Pompeu Fabra (UPF), Barcelona, Spain; 29 University of Manchester & Manchester Royal Eye Hospital, Manchester, UK; 30 ICF, Rockville, MD, USA; 31 National Center for Advancing Translational Sciences, Office of Rare Diseases Research, National Institutes of Health, Bethesda, MD, USA; 32 INSERM, US14—Orphanet, Plateforme Maladies Rares, 75014 Paris, France; 33 The Jackson Laboratory, Bar Harbor, ME, USA; 34 Sanford Imagenetics, Sanford Health, Sioux Falls, SD, USA; 35 Center for Undiagnosed Diseases, Stanford University School of Medicine, Stanford, CA, USA; 36 Department of Genetics, University Medical Center Utrecht, the Netherlands; 37 Institute of Genetic Medicine, Newcastle University, Newcastle upon Tyne, UK; 38 Department of Pediatrics, Stanford University School of Medicine, Stanford, CA, USA; 39 Mount Sinai School of Medicine, New York, NY, USA; 40 Institute of Cardiovascular Science, University College London, UK; 41 Child Neurology Unit. Hospital Universitari Vall d’Hebron, Vall d’Hebron Research Institute (VHIR), Barcelona, Spain; 42 Department of Neuropediatrics and Muscle Disorders, Medical Center—University of Freiburg, Faculty of Medicine, Freiburg, Germany; 43 Children’s Hospital of Eastern Ontario Research Institute, University of Ottawa, Ottawa, Canada; 44 Division of Neurology, Department of Medicine, The Ottawa Hospital, Ottawa, Canada; 45 Centre for Rare Eye Diseases CARGO, SENSGENE FSMR Network, Strasbourg University Hospital, Strasbourg, France; 46 Immunology Service, Department of Laboratory Medicine, NIH Clinical Center, Bethesda, MD, USA; 47 Department of Pediatrics, Division of Allergy Immunology, The Children’s Hospital of Philadelphia, University of Pennsylvania Perelman School of Medicine, 3615 Civic Center Boulevard, Philadelphia, PA 19104, USA; 48 Institute for Systems Genomics, University of Connecticut, Farmington, CT, USA

## Abstract

The Human Phenotype Ontology (HPO)—a standardized vocabulary of phenotypic abnormalities associated with 7000+ diseases—is used by thousands of researchers, clinicians, informaticians and electronic health record systems around the world. Its detailed descriptions of clinical abnormalities and computable disease definitions have made HPO the *de facto* standard for deep phenotyping in the field of rare disease. The HPO’s interoperability with other ontologies has enabled it to be used to improve diagnostic accuracy by incorporating model organism data. It also plays a key role in the popular Exomiser tool, which identifies potential disease-causing variants from whole-exome or whole-genome sequencing data. Since the HPO was first introduced in 2008, its users have become both more numerous and more diverse. To meet these emerging needs, the project has added new content, language translations, mappings and computational tooling, as well as integrations with external community data. The HPO continues to collaborate with clinical adopters to improve specific areas of the ontology and extend standardized disease descriptions. The newly redesigned HPO website (www.human-phenotype-ontology.org) simplifies browsing terms and exploring clinical features, diseases, and human genes.

## INTRODUCTION

A cornerstone of differential diagnostics and translational research is deep phenotyping: the computational analysis of detailed, individual clinical abnormalities (1,2). The Human Phenotype Ontology (HPO) provides the most comprehensive resource for computational deep phenotyping and has become the *de facto* standard for deep phenotyping in the field of rare disease—whether for computable disease definitions, description of clinical abnormalities or to aid genomic diagnostics. A foundational and integrative component of the Monarch Initiative (3,4), the HPO has been adopted internationally by numerous organizations, both academic and commercial; these include the 100,000 Genomes Project, the NIH Undiagnosed Disease Program and Network (UDP and UDN), the Undiagnosed Diseases Network International (UDNI), RD-CONNECT, SOLVE-RD and many others (5–9). The HPO recently achieved status as an International Rare Disease Research Consortium (IRDiRC) recognized resource and is in use by the Global Alliance for Genomics and Health (10) and the associated Matchmaker Exchange (3,11). Here we describe integrated HPO resources which we have revised, expanded, or invented since the previous articles in this series (12,13).

Previously, we reported on a range of algorithms that had been developed by our group and others to support phenotype-driven genomic diagnostics (12). Since then, the HPO has been applied to an increasing range of use cases. Usage of HPO is now commonplace for the analysis of clinical whole-exome and genome sequencing (WES/WGS) data (14–25) as well as for data integration in translational research and bioinformatics (16,26–39). A phenotype risk score based on a mapping of electronic health-record (EHR)-derived billing codes to HPO terms allowed high-throughput ascertainment of EHR phenotypes such that cases and controls of Mendelian diseases could be distinguished and the pathogenicity of variants associated with Mendelian diseases was characterized (40). In another setting, EHR narratives were explored to extract HPO terms by natural language processing and the resulting terms were successfully used to prioritize causal genes for Mendelian diseases in pediatric patients (41). Additionally, an increasing number of commercial applications are using HPO terms. For instance, the SimulConsult Genome-Phenome Analyzer uses HPO terms to tag findings. This is currently being used to document findings entered by the users with codes in exported reports, and the codes will also be used to identify findings in the electronic health record as inputs to be considered in diagnosis (42). A key feature of the HPO is its logical interoperability with basic research ontologies such as the Mammalian Phenotype Ontology (MP) (43), Uberon (44) and the Cell Ontology (45). This interoperability is leveraged within the Exomiser tool (described below). The International Mouse Phenotyping Consortium (IMPC) recently identified 360 new candidate molecular causes of human Mendelian diseases (46); these included an inherited heart disease ‘*Arrhythmogenic Right Ventricular Dysplasia*’ that affects the heart muscle, and *‘Charcot-Marie-Tooth disease’*, which is characterized by nerve damage leading to muscle weakness and an awkward way of walking. This discovery was made possible because (i) the human diseases had been defined in terms of their component HPO phenotypes; (ii) the mouse phenotypes were mapped to the MP; and (iii) Monarch’s phenotype comparison algorithm (47) is designed to traverse HP and MP with ease. Similarly, the Rat Genome Database (RGD) annotates genes, QTLs and strains for phenotype using phenotype terms from the Mammalian Phenotype (MP) Ontology (43); more recently, RGD has converted their annotations of human phenotypes from MP to HPO (48).

HPO has been adopted as the phenotypic annotation ontology of choice for many large-scale rare disease genome-phenome databases and analysis tools including the RD-Connect Genome-Phenome Analysis Platform (GPAP) (49), the Broad Center for Mendelian Genomics and its SEQR platform, the rare disease arm of the UK 100,000 Genomes Project, the NIH Undiagnosed Diseases Program and the Undiagnosed Diseases Network International (UDNI). This is creating a vast body of clinically validated, linked genome-phenome data that not only assists in the diagnosis of the subjects themselves but can be exploited for further developments of the ontology and associated diagnostic algorithms. For example, the RD-Connect GPAP mandates submission of HPO-coded phenotypic data through the PhenoTips tool, using custom-designed disease-specific data collection forms on top of the ‘enter-what-you-see’ HPO entry box. The average number of phenotypic annotations per index case is eight (with an average of six observed and two excluded features) and the GPAP now contains linked genome-phenome datasets on 5000 individuals. Through data submission from European Reference Networks in the Horizon 2020-funded Solve-RD project this number will increase to >20 000 datasets in the coming 2–3 years. The GPAP allows the user to filter variants using predefined gene panels for specific groups of pathologies or alternatively gene lists created ‘on the fly’ based on the HPO terms provided with the individual case. These major databases are not only contributing to gene discovery and diagnosis of the unsolved patients included in the platforms (10) but also providing source data for many computational developments. Within the Solve-RD project (https://solve-rd.eu), RD-Connect worked with Orphanet and HPO to implement the first version of the Phenopackets standard (https://github.com/phenopackets) and export ∼600 cases in Phenopacket format, including clinical phenotype (HPO annotation), clinical diagnosis (ORDO), molecular diagnosis (OMIM) and gene name of genes identified as causal or candidate. The export included both solved cases and unsolved cases that contain sufficient information for phenotypic algorithm evaluation. In addition, work is ongoing that will enable assessment of the correlation between the level, detail and quantity of phenotypic annotation and the solve rate, which will provide clinicians with better advice on the level of detail to provide in their annotations and feed back into improvements to algorithms such as those implemented in Exomiser.

Ontologies should be responsive to the community (43). In the past 2 years we have made improvements to the ontology based on input from clinicians and researchers, as is evidenced by term requests that have been submitted via our GitHub tracker (12). There, we provide a template that guides users through the process of providing information including the suggested term label, definitions and comments, synonyms, references and diseases that should be annotated to the new term. Periodically we also organize collaborative workshops with clinical groups that would like to revise and extend entire areas of the HPO. Five such workshops have been conducted since the 2017 HPO update (Table 1).

**Table 1.** Community workshops and collaborations aimed at HPO content expansion and refinement

Organization	Location	Focus
Undiagnosed Diseases Network (UDN); Stanford Center for Inherited Cardiovascular Diseases (SCICD)	Stanford University, CA, USA (March 2017)	Cardiology
European Reference Network for Rare Eye Disease (ERN-EYE)	Mont Sainte-Odile, France (October 2017)	Ophthalmology
National Institute of Allergy and Infectious Disease (NIAID)	National Institutes of Health, Bethesda, MD, USA (May and July 2018)	Allergy and immunology
Neuro-MIG European network for brain malformations (www.neuro-mig.org)	St Julians, Malta; Lisbon, Portugal (February 2018; September 2018)	Malformations of cortical development (MCD)
European Society for Immunodeficiencies (ESID) and the European Reference network on rare primary immunodeficiency, autoinflammatory and autoimmune diseases (ERN-RITA)	Vienna Austria (September 2018)	Inborn errors of immunity.

The HPO project additionally has a long-term collaboration with Orphanet in the framework HIPBI-RD (harmonizing phenomics information for a better interoperability in the rare disease field), a project that was funded by the E-Rare 3 ERA-NET program (50) and will be continued in the framework of the SOLVE-RD project, as well as in the European Joint Co-fund Programme for Rare Diseases (EJP-RD). This project has resulted in more than 60 000 HPO annotations for diseases in the Orphanet database and over one thousand new term requests and other improvements of existing HPO terms. Phenotype-disease annotations include the frequency of occurrence of a phenotype in a disease (see Table 2), as well as the fact that a phenotype is part of established diagnostic criteria or is a pathognomonic sign. These annotations are available for download and can be consulted in the Orphanet website. Furthermore, this collaboration has produced the HPO-ORDO Ontological Module (HOOM in which the HPO and Orphanet Rare Diseases Ontology can be used together).

**Table 2.** The HPO records the frequencies of phenotypic features in three different ways

Frequency categories		
Term	ID	Definition
Obligate	HP:0040280	Always present, i.e. in 100% of the cases.
Very frequent	HP:0040281	Present in 80–99% of the cases.
Frequent	HP:0040282	Present in 30–79% of the cases.
Occasional	HP:0040283	Present in 5–29% of the cases.
Very rare	HP:0040284	Present in 1–4% of the cases.
Excluded	HP:0040285	Present in 0% of the cases.
*Percentage of persons in which a phenotypic feature is observed*		
Percentage	x%	This is used to record frequency of a feature in a disease if the number of probands is not available, e.g. 42%.
*Number of persons in a cohort in whom a phenotypic feature was observed*		
N of M notation	n/m	This is used to record how many persons with a certain disease were observed to have a given phenotypic feature represented by an HPO term, e.g. 5/13. This should be used only if the feature was ruled out in the remaining m-n individuals.

Frequency information can be used by differential diagnostic algorithms such as BOQA (62). If possible, HPO annotations are made with the precise counts, but percentages or overall frequency categories are used if that is all that is available. The frequency categories are aligned with those of Orphanet.

## LOGICAL ENHANCEMENTS AND INTEROPERABILITY

The HPO provides textual definitions for ease of use, but it also has a robust logical representation with OWL-based logical definitions based on species-neutral ontologies such as Uberon, the Gene Ontology, the Cell Ontology and others. For instance, *Delayed patellar ossification* (HP:0006454) is defined with reference to the PATO term *delayed* (PATO:0000502), the Gene Ontology term *ossification* (GO:0001503) and the Uberon term for *patella* (UBERON:0002446). The OBO version of the ontology is a simplified version of the full OWL version that contains all of the terms as well as their subclass (is-a) relations, but does not contain the computational logical definitions.

‘has part’ some

(delayed

and (‘inheres in’ some

(ossification

and (‘occurs in’ some patella)))

and (‘has modifier’ some abnormal))

These logical definitions can be used for quality control (51), to infer new classifications (is_a/subclass relationships) that were not explicitly asserted and for cross-species phenotype analysis (46). However, this can only work if compatible sets of definitions are used.

Manually maintaining compatible logical definitions across large ontologies such as the HPO is error-prone and may lead to inconsistent description in one ontology and especially across different phenotype ontologies. Even specialized branches of the ontology, such as the ones addressing morphological abnormalities, can have divergent logical definitions. Pattern-based ontology development practices (52,53) are increasingly used to manage the generation of logical definitions. Rather than encoding logical definitions manually in OWL using an ontology editor, pattern-based development separates the blueprint of the logical definition—essentially the definition with placeholder variables—from the actual definition of the term, which is usually encoded in the form of a spreadsheet record. Members of the Monarch Initiative are contributing to community tools for pattern-based development using Dead Simple Ontology Design Patterns (DOSDP, (52)) and the Ontology Development Kit (ODK).

To support the use of model organisms to further human health research, developers of the Mammalian Phenotype (MP) ontology (54) have collaborated with the HPO team to develop compatible logical definitions, but these efforts were restricted to comparison of individual definitions and resulted in manual changes to the respective ontologies. Pattern-based development offers a more accurate and scalable alternative by developing common patterns that all phenotype ontologies (i.e. all organisms) can refer to and that can be applied to a whole branch of an ontology at once. For example, the ‘increasedSize’ pattern defines a blueprint for a logical definition as follows: ‘‘has_part’ some (‘increased size’ and (‘inheres_in’ some %s) and (‘qualifier’ some ‘abnormal’))’. Using DOSDP in conjunction with the ODK, any phenotype ontology developer who needs to define a phenotype describing the increased size of something (such as an anatomical entity) can now simply commit to the increasedSize pattern. More than 40 patterns specifically for phenotype ontology development are currently available in the Uber-Phenotype (UPheno) repository.

The clinical features represented in HPO are connected via subclass relations. Other relationships between those classes hold as well, but have not previously been encoded computationally. For example, phenotype ontologies may have two separate classes to represent the increase and decrease in size of an anatomical entity such as the liver. To represent such relations, we have added opposite relations to all terms in HPO using a text and logic-based approach (see phenopposites GitHub repository under ‘Availability’).

The Monarch Initiative has been a key organizer of a community effort to use pattern-based ontology development to reconcile logical definitions on a large scale across well-established and emerging phenotype ontologies including HPO, MP, and phenotype ontologies for *Caenorhabditis elegans, Xenopus* and *Drosophila*. To that end, we recently organized a Phenotype Ontology development and reconciliation workshop (Phenotype Ontologies Traversing All The Organisms: POTATO). At this workshop, more than 40 ontology curators, developers and biomedical experts came together to learn about our updated tool-chain for pattern-based development and to discuss discrepancies between the logical definitions across various phenotype ontologies. As a result of the meeting, representatives of all the phenotypes ontologies have committed to an ongoing collaboration to align their respective ontologies by developing sets of common design patterns and using these to define terms in their ontologies. The outcome of these community efforts will be an integrated ecosystem of phenotype ontologies that can be leveraged in HPO-based clinical diagnostics and disease mechanism discovery.

## DISEASE ANNOTATIONS

The HPO project provides a comprehensive set of computable definitions of rare diseases in the form of annotations which describe the clinical features (HPO terms) that characterize each disease. Each annotated feature can have metadata including its typical age of onset and the frequency (for instance, the HPO lists the frequency of *Protrusio acetabuli* [HP:0003179] in persons with Marfan syndrome as 113/146 based on a published clinical study (55)). Such annotation metadata can be used to improve the accuracy of the HPO-based matching algorithms (56).

Recent updates to our corpus of disease annotations include a new file format with robust representation of clinical modifiers, as well as migration to the Monarch Merged Disease Ontology (MONDO), which provides a unified set of disease terms and definitions with computationally declared equivalencies to resources such as OMIM and Orphanet. The annotation data is readily available for computational use via Monarch’s Biolink API (see resources below). We have also produced a new stand-alone tool to aid curation of the disease annotations.

Thirty-six new molecular phenotypes have been added to the HPO. These new terms were identified from metabolomics data provided by the Metabolomics Core from the Undiagnosed Disease Network, the Human Metabolome Database (HMDB) and articles related to inborn errors of metabolism. The new terms were curated in a spreadsheet that captured information about metabolite name, corresponding chemicals and their identifiers (ChEBI and HMDB), direction of change (increase/decrease), location of the abnormal metabolite concentration (blood, urine, cerebrospinal fluid), synonyms, gene/locus association, disease identifiers for associated diseases (OMIM or MONDO IDs) and key publication (PubMed IDs). For instance, an *increased level of galactonate in red blood cell* (HP:0410063) is associated with patients with galactosemia (MONDO:0018116; gene: *GALT*).

**Figure 1.**
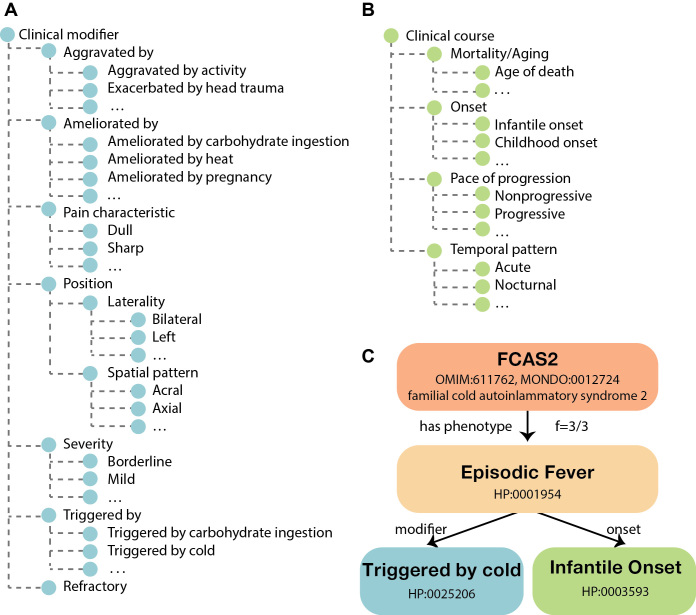
Overview of the **clinical modifier** (**A**, left) **and clinical course** (**B**, right) subontologies. These subontology terms can be used in combination with existing HPO terms to qualify and enrich their meaning. (**C**) A schematic presentation of one HPO annotation for the disease familial cold autoinflammatory syndrome 2 (FCAS2). In a publication on this disease, three of three reported patients were found to have episodic fever with infantile (or earlier) onset that was triggered by exposure to cold (63).

The new *Clinical modifier* subontology allows more expressive and precise disease definitions and can also be used to annotate individual patients. This subontology contains terms to describe severity, positionality and external factors that tend to trigger or ameliorate the features of a disease. The previous Onset subontology has been expanded to a Clinical course subontology, which additionally contains terms to describe mortality, progression of disease and the temporal pattern of features of disease (Figure 1). The frequency of features can be described in one of three methods (Table 2).

**Figure 2.**
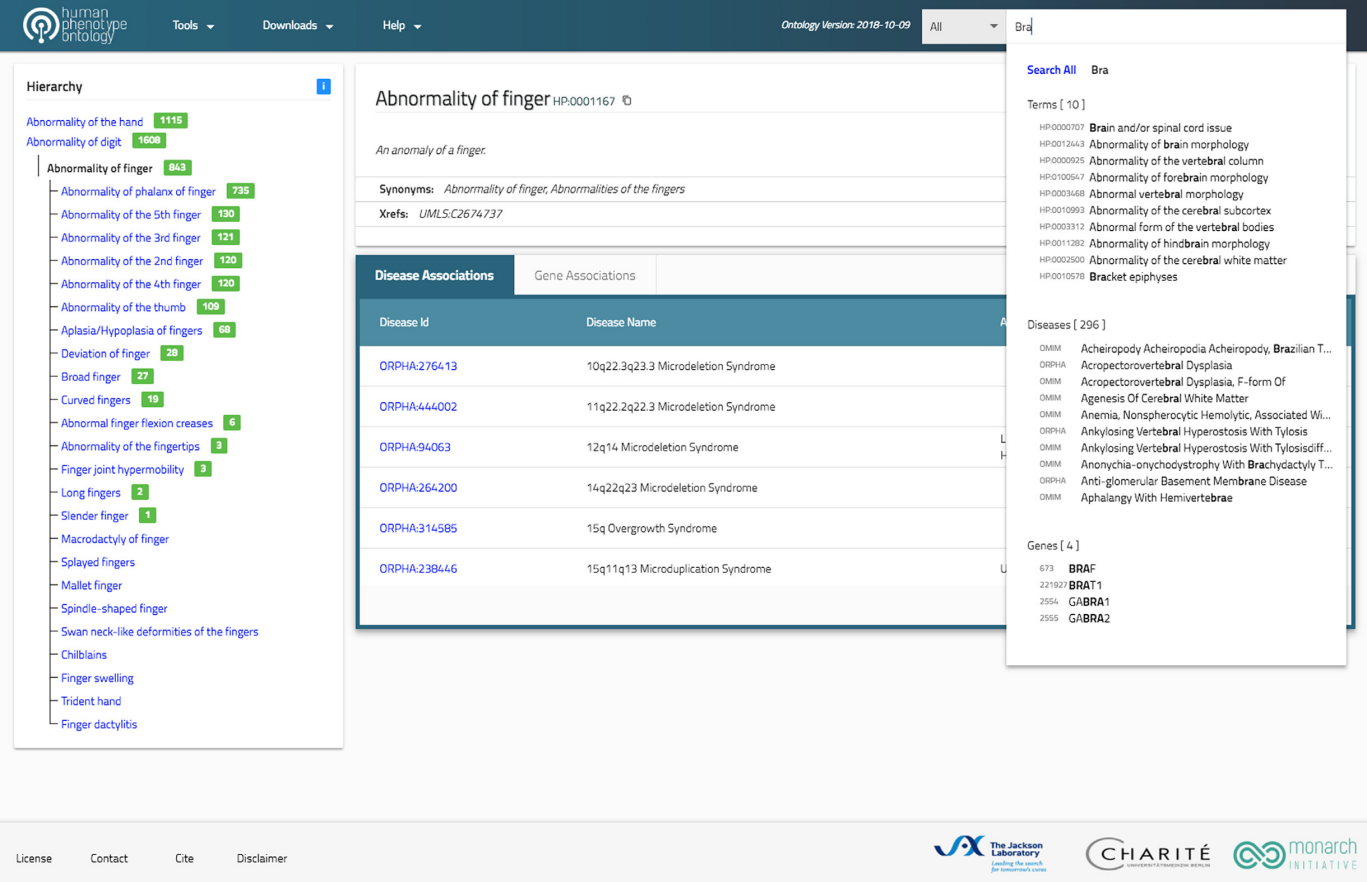
Screenshot of the new HPO Website application. Users can search for HPO terms, annotated diseases, or disease-associated genes using an autocomplete widget. The hierarchical structure of the ontology is shown in an abbreviated fashion for clarity’s sake. Only the direct parent and child terms of the currently displayed term are shown in the hierarchy. The total number of decedent terms is shown for each term in the hierarchy to help users decide which parts of the ontology to explore.

The HPO annotation file format had remained unchanged since the first publication of the HPO in 2008 (57); to accommodate the aforementioned new annotation resources, we have updated the annotation file format. This format has slots to capture clinical modifiers, sex-specific features of disease and to track the history of biocuration of terms (Table 3).

**Table 3.** New HPO annotation file format

Field	Item	Required	Example
1	Database ID	Yes	MIM:154700, ORPHA:558 or MONDO:0007947
2	DB_Name	Yes	Achondrogenesis, type IB
3	Qualifier	No	NOT or empty
4	HPO_ID	Yes	HP:0002487
5	DB_Reference	Yes	OMIM:154700 or PMID:15517394
6	Evidence	Yes	IEA
7	Onset	No	HP:0003577
8	Frequency	No	HP:0003577 or 12/45 or 22%
9	Sex	No	MALE or FEMALE
10	Modifier	No	HP:0025257
11	Aspect	Yes	‘P’ or ‘C’ or ‘I’ or ‘M’
12	BiocurationBy	Yes	HPO:skoehler[YYYY-MM-DD]

The file contains 12 tab-separated fields, some of which can be left empty. The ‘Modifier’ and ‘BiocurationBy’ fields can contain multiple items separated by semicolons. For instance, to indicate that a disease is characterized by a skin rash (HP:0000988) that is Recurrent (HP:0031796) and Triggered by cold (HP:0025206) one would annotate HP:0031796;HP:0025206 in the Modifier column. Many annotations go through multiple stages of biocuration. In this case, the individual biocuration events are also added as a semicolon-separated list.

A new tool called HPOWorkbench has been developed to enable browsing through HPO terms and annotations. It can generate GitHub issues directly and can be used by collaborators to provide feedback or new suggestions.

## EXOMISER UPDATE

Exomiser utilizes the HPO to find potential disease-causing variants from whole-exome or whole-genome sequencing data. The last two major updates to the Exomiser software have focused on decoupling the data updates from the software release cycle and enabling analysis of either GRCh37 or GRCh38 genomic samples. We updated the variant data sources to also include allele frequency data from gnomAD, TOPMed and the UK10 datasets and added annotations for variant pathogenicity from ClinVar. We also added the ability for users to specify fine-grained maximum allele frequencies to be used for prioritizing alleles under different inheritance models and assigning these to likely syndromes based on the phenotype matches. Moreover, the Exomiser variant data sources have not only been decoupled from the software release cycle, but also from the phenotype ontologies and disease annotations. This ensures that we can release Exomiser with the very latest disease and model organism annotations and that they can be updated on demand. These user-facing updates have happened against a background of continued engineering and performance improvements. As a result of the continued development and usage, the Exomiser also recently received the approval of the International Rare Diseases Research Consortium (IRDiRC) as a recognized resource. We have also been able to build on HPO being chosen as the terminology for clinical phenotype data collection by the UK National Health Service (NHS) by introducing Exomiser as a key variant prioritization service for the 100 000 Genomes Project and future NHS-commissioned service for rare disease genetic testing. Benchmarking on the solved cases to date shows Exomiser can identify over 80% of the diagnoses in the top five candidates (unpublished communication from the 100K Genome project).

## SYNONYMS AND TRANSLATIONS

One of the key advantages of ontologies is that semantic meaning is attached to concepts, rather than to their names. This enables each entity to have one or more synonyms, as well as translations into other languages. Multiple groups have taken advantage of this ability to create synonyms for HPO concepts for diverse settings, including enabling self-phenotyping by patients without medical expertise and enabling capture of data in diverse languages, with subsequent international sharing and analysis.

Patients themselves are an eager and untapped source of information about symptoms and phenotypes, however, medical terminology is often perplexing to them, making it difficult to use resources like the HPO. Further, some phenotypes go unnoticed by the clinician (such as those only seen at home). To enable patients to use the HPO directly and to improve collaboration and communication between patients and their physicians, we have recently added ‘layperson’ synonyms to the entirety of the HPO (58). Approximately 36% of the HPO terms have at least one layperson synonym, 89% of the MONDO diseases annotated to HPO have at least one HPO annotation with a layperson synonym and 60% of all disease annotations refer to HPO terms with lay translations. This coverage suggests that the layperson HPO would be useful in a diagnostic setting despite incomplete coverage. Efforts are currently underway to evaluate the diagnostic utility of the layHPO, both synthetically as well as in cohorts of previously diagnosed rare disease patients.

The Sanford Health Imagenetics program has deployed an online screening tool for patients to self-report traits, signs, and symptoms in a questionnaire format that is mapped to HPO and leverages the layperson synonyms. This is integrated with the Sanford Imagenetics population-based genotyping initiative. The Genetic and Rare Diseases Information Center (GARD), a program of the National Center for Advancing Translational Sciences Office of Rare Diseases Research (NCATS-ORDR), provides reliable, public-friendly information for over 7000 genetic and/or rare diseases (59). GARD recently incorporated tables on the disease webpages that display information from the HPO including the medical terms for associated symptoms and phenotypic abnormalities, the related layperson synonyms, the frequency of the phenotypic features and the link to the HPO webpage for the specific term. By displaying the plain-language vocabulary along with the medical terminology, patients and families become familiar with the language they are commonly exposed to in the literature and clinical settings. The public utilizes the HPO medical terms and layperson synonyms to better understand the broad spectrum of clinical findings associated with a specific disease and to search and navigate the GARD website and other resources to retrieve information about multiple diseases associated with a given phenotype. Inclusion of the HPO data on the GARD website makes the disease webpages more robust, educates the rare disease community and empowers them to become partners in their medical care.

The labels, synonyms and textual definitions of the HPO are also being translated into several languages including French, Spanish, Italian, German, Dutch, Portuguese, Turkish, Japanese, Russian and Chinese; this is critical to ensure equitable health care and precision public health (See project homepage below). Tools such as PhenoTips (60) already make use of the existing Spanish and French translations, together with a user interface in those languages to enable HPO-based phenotyping for clinicians who are not fluent in English. In the Spanish Undiagnosed Disease Network clinicians phenotype patients in Spanish, and then share with the Matchmaker Exchange (13). One further example is the Life Languages project in Western Australia (WA), which is using the HPO to translate medical and biological terms into partner Aboriginal Australian Languages. This is being integrated with HPO term extraction from 3D facial images as part of the Pilbara Faces program in remote WA.

## NEW HPO WEBSITE

The HPO website application has been redesigned and rebuilt from the ground up to be both more responsive and more intuitive (Figure 2). Made possible by the new single-page app approach and lightweight microservices, the new application loads faster and supports intuitive search capabilities, such as auto-complete and term highlight features, to allow the user to efficiently browse through the ontology data and corresponding hierarchy. The HPO website uses the ProtVista tool to display genes and genetic variants associated with Mendelian diseases (61). The redesign also sets the stage for better integration with monarchinitiative.org to facilitate exploration of similar genes and phenotypes across species.

## HPO FOR MEDICAL EDUCATION

Clinical features in HPO are also connected to disease nosologies (medical classification schemes) such as ORDO, OMIM, and MONDO. These relationships are typically curated from literature; however, they can also be crowd-sourced. Phenotate (http://phenotate.org), which was developed in the framework of the HIPBI-RD project, is a web-based tool that allows undergraduate or medical students, as well as medical residents, to annotate OMIM and ORDO diseases with HPO phenotypes by completing classroom exercises. Students are encouraged to refer to the literature to select the correct symptoms and enter the references used into their annotations. In a second-year undergraduate molecular genetics class (MGY200) at the University of Toronto, 78 students used Phenotate to annotate three genetic diseases: Marfan syndrome (MFS), Friedreich’s ataxia (FRDA) and congenital myasthenic syndrome. Overall, students collectively provided more comprehensive annotations than clinicians who also submitted annotations. Phenotate is an open platform, available for use by anyone teaching genetics. By crowdsourcing annotations, Phenotate hopes to improve the HPO and related nosologies, while also offering students an educational tool that supplements their coursework.

## CONCLUSION

In the 2 years since the previous Nucleic Acids Research database article (12), the HPO has continued to grow in both reach and scope. The HPO has put a strong emphasis on working with interested members of the community to revise and extend individual areas of the HPO, and we welcome interactions with more groups in any area of medicine. The HPO project has begun to develop resources for laypersons to interact with the HPO and software designed for patients. Annotations and improved representation of phenotypes in the HPO have been greatly improved for several areas of medicine thanks to community interactions.

## DATA AVAILABILITY

The Human Phenotype Ontology and its resources are available at http://www.human-phenotype-ontology.org
Web version of the Exomiser: https://phenomics-dev.kccg.garvan.org.au/web-exomiser/
Orphanet: http://www.orpha.net
HOOM: HPO-ORDO Ontological Module: http://www.orphadata.org/
Biolink: https://api.monarchinitiative.org/api/
MONDO: http://obofoundry.org/ontology/mondo.html
Monarch Initiative: https://monarchinitiative.org
Phenotate: http://phenotate.org
HPOWorkbench: https://github.com/TheJacksonLaboratory/HPOworkbench
Ontology Development Kit: https://github.com/INCATools/ontology-development-kit
UPheno: https://github.com/obophenotype/upheno
Phenopposites: https://github.com/Phenomics/phenopposites
HPO Translation Project: https://crowdin.com/project/hpo-translation
